# Presence of *qnr *gene in *Escherichia coli *and *Klebsiella pneumoniae *resistant to ciprofloxacin isolated from pediatric patients in China

**DOI:** 10.1186/1471-2334-8-68

**Published:** 2008-05-22

**Authors:** Aihua Wang, Yonghong Yang, Quan Lu, Yi Wang, Yuan Chen, Li Deng, Hui Ding, Qiulian Deng, Hong Zhang, Chuanqing Wang, Lan Liu, Xiwei Xu, Li Wang, Xuzhuang Shen

**Affiliations:** 1 Beijing Children's Hospital, Affiliated to Capital Medical University, Beijing, PRoC; 2 Shanghai Children's Hospital, Affiliated to Shanghai Jiao Tong University, Shanghai, PRoC; 3 The Children's Hospital of Fudan University, Shanghai, PRoC; 4 Chongqing Children's Hospital Affiliated to Chongqing Medical University, Chongqing, PRoC; 5 Guangzhou Children's Hospital, Affiliated to Guangzhou Medical College, Guangzhou, PRoC

## Abstract

**Background:**

Quinolone resistance in *Enterobacteriaceae *results mainly from mutations in type II DNA topoisomerase genes and/or changes in the expression of outer membrane and efflux pumps. Several recent studies have indicated that plasmid-mediated resistance mechanisms also play a significant role in fluoroquinolone resistance, and its prevalence is increasing worldwide. In China, the presence of the *qnr *gene in the clinical isolates of *Enterobacteriaceae *has been reported, but this transmissible quinolone resistance gene has not been detected in strains isolated singly from pediatric patients. Because quinolones associated with a variety of adverse side effects on children, they are not authorized for pediatric use. This study therefore aimed to investigate the presence of the *qnr *gene in clinical isolates of *E. coli *and *K. pneumoniae *from pediatric patients in China.

**Methods:**

A total 213 of non-repetitive clinical isolates resistant to ciprofloxacin from *E. coli *and *K. pneumoniae *were collected from hospitalized patients at five children's hospital in Beijing, Shanghai, Guangzhou, and Chongqing. The isolates were screened for the plasmid-mediated quinolone resistance genes of *qnrA*, *qnrB*, and *qnrS *by PCR. Transferability was examined by conjugation with the sodium azide-resistant *E. coli *J53. All *qnr*-positive were analyzed for clonality by enterobacterial repetitive intergenic consensus (ERIC)-PCR.

**Results:**

The study found that 19 ciprofloxacin-resistant clinical isolates of *E. coli *and *K. pneumoniae *were positive for the *qnr *gene, and most of the *qnr *positive strains were ESBL producers. Conjugation experiments showed that quinolone resitance could be transferred to recipients. Apart from this, different DNA banding patterns were obtained by ERIC-PCR from positive strains, which means that most of them were not clonally related.

**Conclusion:**

This report on transferable fluoroquinolone resistance due to the *qnr *gene among *E. coli *and *K. pneumoniae *strains indicated that plasmid-mediated quinolone resistance has emerged in pediatric patients in China.

## Background

Quinolone resistance in *Enterobacteriaceae *results mainly from mutations in type II DNA topoisomerase genes[1] and/or changes in the expression of outer membrane and efflux pumps[2]. Recently, studies have shown that plasmid-mediated resistance mechanisms also play a significant role in fluoroquinolone resistance, and its prevalence is increasing worldwide [3]. *qnrA *is the plasmid-mediated quinolone resistance gene encoding a 218 amino acid protein of the pentapeptide family that protects DNA gyrase from quinolone inhibition[4]. The new plasmid-mediated quinolone resistance genes, *qnrB *and *qnrS*, have been reported in clinical isolates[5,6]. In China, the presence of the *qnr *gene in clinical isolates of *Enterobacteriaceae *from Shanghai [7] and Anhui[8] has been reported, but this transmissible quinolone resistance gene has not been detected in strains isolated singly from pediatric patients. Because quinolones associated with a variety of adverse side effects on children, they are not authorized for pediatric use. Therefore, the objective of this study was to screen for the presence of the *qnr *gene in clinical ciprofloxacin-resistant isolates of *Escherichia coli *and *Klebsiella pneumoniae *from pediatric patients in China.

## Methods

### Bacterial strains

Three hundred thirty-five *Escherichia coli *and 392 *Klebsiella pneumoniae *non-replicate clinical isolates were collected from five children's hospitals located in Beijing, Shanghai, Guangzhou, and Chongqing from January 2005 to December 2006. Each hospital is a general pediatric hospital locally affiliated to a university. The isolates were identified at the participating hospitals by routine methodology at each laboratory, and then were transported to Beijing Children's Hospital for further analysis. All isolates were screened for ciprofloxacin resistance by disk method according to the criteria of NCCLS [9]. The screening showed 146 *Escherichia coli *and 67 *Klebsiella pneumoniae *isolates were ciprofloxacin-resistant. Additional strain used was *E. coli *J53Az^R^(resistant to azide) as a recipient of the conjugation experiment

### Screening for the *qnrAM*, *qnrB*, and *qnrS *gene in clinical strains

The 213 ciprofloxacin-resistant strains of *E. coli *and *K. pneumoniae *were screened by multiplex PCR amplification of *qnrA, qnrB*, and *qnrS *as previously described[10]. Colonies were transferred to an Eppendorf tube filled with water and boiled to prepare DNA templates for PCR. The primers used for *qnrA, qnrB, and qnrS *were as follows: '5-ATTTCTCACGCCAGGATTTG-3' and 5'-GATCGGCAAAGGTTAGGTCA-3' for *qnrA *for a 516-bp product, 5'-GATCGTGAAAGCCAGAAAGG-3' and 5'-ACGATGCCTGGTAGTTGTCC-3' for *qnrB *for a 469-bp product, and 5'-ACGACATTCGTCAACTGCAA-3' and 5'-TAAATTGGCACCCTGTAGGC-3' for *qnrB *for a 417-bp product. The protocol for the PCR condition was: 94°C for 45 s, 53°C for 45 s, and 72°C for 60 s, with a cycle number of 32, and without DNA template as negative controls in each run. Amplification products were provisionally identified by their size in ethidium bromide-stained agarose gels. The positive amplified PCR product for *qnrA, qnrB*, and *qnrS *was analyzed with an automated DNA sequencing system. The results of DNA sequences were compared with the BLAST online search engine from GenBank at the National Center for Biotechnology Information Web site: .

### Conjugation experiments

All positive *qnr *strains were tested for transferred quinolone resistance by conjugation experiments carried out in Luria Broth with *E. coli *J53Az^R ^as the recipient. The cultures of donor and recipient cells were incubated via shaking at 37°C in logarithmic phase, and then 0.5 mL of both cultures was added to 4 mL of fresh LB and incubated overnight without shaking. Transconjugants were selected on trypticase soy agar (TSA) plates with sodium azide (100 μg/mL from Sigma Chemical Co., St. Louis, MO) for counter selection and ampicillin (100 μg/mL from Oxoid) to select for plasmid-encoded resistance. To determine if quinolone resistance was cotransferred, colonies were replica-plated onto TSA with and without ciprofloxacin (0.06 μg/mL; Oxoid). The PCR experiments confirmed transconjugants carrying the same *qnr *gene as their donors.

### Antimicrobial susceptibility profiles

MICs for the 19 positive *qnr *isolates, recipient (J53 Az^R^), and transconjugant strains were measured by agar dilution in accordance with the guidelines of the NCCLS. The antimicrobials tested were ampicillin/clavulanic acid, cefotaxime, ceftazidime, cefoperazone, cefoxitin, cefepime, aztreonam, imipenem, ciprofloxacin, ofloxacin, amikacin, and gentamicin (Oxoid, England). Quality control was performed by testing *Escherichia coli *ATCC25922. Isolates which showed MICs ≥ 2 μg/ml for cefotaxime and/or ceftriaxone and/or ceftazidime and/or aztreonam were considered as ESBL producers. The ESBL phenotype was confirmed by using clavulanic acid (Oxoid, England) according to the manufacturer's recommendation.

### Strain typing by ERIC-PCR

The 11 positive *qnr Escherichia coli *strains and 8 positive *qnr Klebsiella pneumoniae *strains were typed by ERIC-PCR. Total DNA from *qnr *positive isolates was analyzed by ERIC sequence PCR with the ERIC1R (*5'-ATGTAAGCTCCTGGGGATTCAC-3'*) and ERIC2 (*5'-AAGTAAGTGACTGGGGTGAGCG-3'*) primers, and PCR was performed as previously described [11]. ERIC-PCR was performed briefly using the following program parameters: denaturation at 94°C for 1 s, annealing at 52°C for 10 s, and extension at 72°C for 35 s for 30 cycles, followed by a final extension at 72°C for 4 min. Amplicons were separated on a 1.5% agarose gel containing ethidium bromide (5 μg/mL) at 60 V for 3 hours. The gels were photographed and digitized. Isolates were considered different if their profiles differed by two or more bands[12].

## Results

### Screening for the qnr genes

Nineteen ciprofloxacin-resistant clinical isolates, including eleven *E. coli *and eight *K. pneumoniae *strains, were positive for the *qnr *gene. Table 1 shows the clinical characteristics of these isolates and the distribution of *qnrA*, *qnrB*, or *qnrS*. The sequences of *qnr *genes were all identical to those of *qnrA *(EU195836), *qnrB *(EU443840), and *qnrS *(EU391634) with GenBank, respectively.

**Table 1:** Clinical characteristics and *qnr *genotype of the *qnr*-positive isolates

Number of Strains	Specimen	Sex	Age	Diagnoses	*qnr*	ESBL
*E. coli*						
						
05B239	sputum	m	2 months	Pneumonia	*qnrA, qnrB, qnrS*	-
05C2795	sputum	f	15 days	Pneumonia	*qnrA*	+
						
05G1844	vulvar secretions	f	5 years	Vulvitis	*qnrA, qnrS*	-
05G1889	urine	m	2 months	Hydronephrosis	*qnrA, qnrB*	+
05SB14	sputum	f	24 days	Pneumonia	*qnrS*	+
05SB25	urine	m	5 months	UTI^a^	*qnrA, qnrB*	+
05SB47	tracheal	f	12 months	Pneumonia	*qnrA, qnrB*	+
06G60	blood	m	3 years	ALL^b^	*qnrA*	+
06G62	sputum	f	24 days	Pneumonia	*qnrS*	+
						
06G99	sputum	f	4 months	Pneumonia	*qnrA, qnrB*	-
06SA30	sputum	f	2 months	Pneumonia	*qnrS*	+
*K. pneumoniae*						
05C2978	sputum	m	9 months	Pneumonia	*qnrB*	+
05G44	sputum	f	1 months	Pneumonia	*qnrB*	+
05SA32	sputum	f	21 months	Pneumonia	*qnrS*	+
06B295	sputum	m	11 days	Pneumonia	*qnrB*	+
06B700	urine	f	4 years	UTI^a^	*qnrB*	+
06C3889	sputum	f	4 days	Pneumonia	*qnrS*	+
06C5524	sputum	f	3 months	Pneumonia	*qnrB*	+
06SB60	sputum	m	3 months	Pneumonia	*qnrS*	+

^a^UTI, Urinary tract infection;

^b^ALL, Acute lymphoblastic leukemia.

### Conjugation and antimicrobial susceptibility

Quinolone resistance could be transferred by conjugation in two of eight of *Klebsiella pneumoniae qnr*-positive donors. One *qnrB*-positive and one *qnrS*-positive transconjugant were obtained. PCR experiments confirmed that the transconjugants harbored the same *qnr *gene as their donors. Table 2 shows the susceptibilities of the *qnr*-positive clinical isolates for the selected antibiotics. Most isolates were resistant to cephalosporin, aztreonam, and aminoglycosides, whereas all of the strains were susceptible to imipenem. Eight of eleven *qnr *positive isolates of *Escherichia coli *and all eight *qnr *positive isolates of *Klebsiella pneumoniae *were phenotypic ESBL producers, respectively. Antibiotic susceptibilities for the two transconjugants showed that the ciprofloxacin resistance MIC value increased 32- and 1024-fold, respectively; meanwhile, oflaxacin resistance MIC value increased 2- and 32-fold, respectively.

**Table 2:** Resistance profiles of the *qnr *positive strains and transconjugants

Strain	MIC(μg/ml)											
	
	AMC^a^	CFP	CTX	CAZ	FOX	FEP	IPM/CS	ATM	AMK	GEN	CIP	OFX
*E. coli*												
05B239	32	> 512	512	32	32	512	0.25	64	4	128	64	32
05C2795	128	512	64	256	512	16	0.5	256	16	256	16	32
05G1844	32	512	64	8	32	8	0.125	16	4	128	64	32
05G1889	32	512	> 512	32	32	128	0.25	32	8	128	64	64
05SB14	16	> 512	128	8	32	16	0.125	8	8	256	64	16
05SB25	32	> 512	> 512	128	32	128	0.125	64	4	128	64	32
05SB47	32	> 512	> 512	512	16	> 512	0.25	256	8	128	256	64
06G60	32	512	512	32	16	128	0.25	64	4	128	32	16
06G62	16	512	512	8	32	32	0.125	8	8	128	64	32
06G99	64	> 512	> 512	512	512	128	0.25	256	4	128	64	64
06SA30	32	512	> 512	8	32	32	0.25	16	16	256	4	32
*K. pneumoniae*												
05C2978^b^	32	128	128	512	16	64	0.125	128	4	32	256	128
05SA32^b^	32	256	256	512	16	128	0.125	128	8	2	4	8
05G44	128	> 512	> 512	> 512	512	512	1	512	8	32	4	4
06B295	32	> 512	128	64	16	64	1	64	8	2	4	8
06B700	32	512	512	256	256	128	0.25	128	8	128	32	8
06C3889	32	> 512	256	512	32	64	0.125	256	> 512	> 512	256	256
06C5524	32	256	128	512	16	64	0.5	512	> 512	512	64	32
06SB60	32	> 512	> 512	64	32	512	0.25	256	4	128	4	32
Recipients												
J53	16	2	0.125	0.25	2	1	0.0625	4	8	0.25	0.125	0.25
Transconjugants												
Tc 05C2978^c^	32	128	128	512	64	32	0.125	128	8	512	128	8
Tc^b ^05SA32^c^	32	512	512	512	128	256	0.25	128	16	128	4	0.5

^a ^AMC, amoxicillin/clavulanic acid; CFP, cefoperazone; CTX, cefotaxime; CAZ, cefotaxime; FOX, cefoxitin; FEP, cefepime; IPM/CS, imipenem/cilastatin; ATM, aztreonam; AMK, amikacin; GEN, gentamicin; CIP, ciprofloxacin; OFX, ofloxacin.

^b ^domor;

^c ^Tc, transconjugants

### Strain typing by ERIC-PCR

The positive qnr isolates of *E. coli*, and *K. pneumoniae *showed different DNA banding patterns, indicating that they were not clonally related.

## Discussion

This study is the first report on the presence of the *qnr *gene in clinical isolates from pediatric patients in China. The prevalence rates of *qnr *among ciprofloxacin-resistant isolates of *E. coli *and *K*. were 7.5% (11 of 146), and 11.9% (8 of 67), respectively. The *qnr *rate in isolates of *Escherichia coli *analyzed in this study was similar to the prevalence in Shanghai, China, wherein there was 8% (6 of 78 strains) of ciprofloxacin-resistant clinical isolates of *E. coli *[7]. These rates are lower than the prevalence of *qnrA *detected among ceftazidime-resistant or ciprofloxacin-resistant strains (24%) of *Enterobacter *in USA[13]. Among the three groups of the *qnr *gene, *qnrA *was more prevalent (8 of 11) in *Escherichia coli *strains, whereas *qnrB *was more prevalent (5 of 8) in the *Klebsiella pneumoniae *isolates. As there are differences in the criteria for testing strains, evaluating the precise prevalence of plasmid-mediated quinolone resistance was difficult.

It should be noted that all the positive *qnr *isolates were distributed in pediatric patients: more than one-third were isolated from children younger than one year of age, and nearly one-fourth were isolated from neonates. Because of the variety of adverse side effects of quinolones in children, hospitals are not advised to use them on children. In China, quinolones are not used on children younger than 16 years old. Presumably, the source of the *qnr *gene might not be directly associated with the selective pressure caused by the quinolones used in pediatrics, but it could be related to horizontal transmission from adults or other reservoir. Recent findings showed that these genes come from environmental gram-negative bacterial species, such as *Shewanella algae*, the progenitor of the *qnrA *genes [14], and *Vibrio splendidus *or *Aeromonas spp*., the progenitor of *qnrS *genes[15,16]. This shows that the aquatic environment is an important reservoir of novel antibiotic resistance determinants. Quinolones are antimicrobial agents extensively used in aquaculture and are stable molecules in water [17]. Exposure to lower concentrations of quinolones increases the chance for seletion of resistance. Therefore, they may be the source of an important driving force for the selection of quinolone resistance. Another study showed that *qnrS *genes were identified from *E. coli *and *E. cloacae *isolated from zoo animals in Japan, suggesting that animals could be a potential reservoir of quinolone-resistant bacteria[18]. Quinolones are the most common antimicrobial used in animals, the annual consumption of which is about 470 tons in China [19]. In the present study, more than two-thirds of *qnr *positive strains were isolated from sputum sample. These strains may be confined in children's respiratory tract, which come from the contaminated environment.

This study showed that most *qnr *positive strains were ESBL producers, indicating the relationship between the *qnr *gene and ESBL. Other studies have shown that several *qnrA*-positive isolates express ESBL [7,20,21]. A *qnrS *gene on a plasmid encoding ESBL in a *K. pneumoniae *strain also has been reported in Taiwan, China [22]. Therefore, the fact that expanded-spectrum cephalosporins are widely used among Chinese children may also contribute to the presence of the *qnr *gene. Another study by the present researchers suggested that third-generation cephalosporins are commonly used in pediatric patients (data not published).

Conjugation experiments showed that only two *qnr*-positive *K. pneumoniae *strains were able to transfer the *qnr *gene in transconjugants. This transferability was similar to the findings of other studies, which also showed that not all *qnr*-positive strains were able to transfer quinolone resistance [23,24]. The lower resistance to ciprofloxacin and oflaxacin in these two transconjugants than their donor strains may imply the presence of additional chromosomal resistance mutations. The results of ERIC-PCR showed that the positive *qnr *strains have different types of DNA band, thereby suggesting that the *qnr *in clinical isolates was not caused by the spread of identical strains.

Although the number of data points is limited, this report confirms the spread of the plasmid-mediated quinolone resistance *qnr *gene in *E. coli *and *K. pneumoniae *strains isolated from pediatric patients. Still, however, further research is needed to understand the influence of this gene on quinolone resistance for pediatric patients in China.

## Conclusion

The prevalence rates of *qnr *among the clinical isolates of ciprofloxacin resistance in *E. coli *and *K. pneumoniae *were 7.5% (11 of 146) and 11.9% (8 of 67), respectively. The transferability of fluoroquinolone resistance because of the *qnr *gene among *E. coli *and *K. pneumoniae *strains shows that plasmid-mediated quinolone resistance has been spread in pediatric patients in China.

## Competing interests

The authors declare that they have no competing interests.

## Authors' contributions

All authors of the present study contributed substantially to the conception and design of the study, were involved in the writing and in the critical revision of the manuscript, and read and approved the final version of the manuscript submitted for publication. AW was mainly responsible for data analysis and preparation of the manuscript; XS and YY conceived of the study, participated in its design and coordination, and helped in drafting the manuscript; QL, YW, YC, LD, QD, HZ, CW, LL, and XX were mainly responsible for the collection of clinical isolates and information about the patients; and HD and LW facilitated the PCR and conjugation experiments on the samples.

## Pre-publication history

The pre-publication history for this paper can be accessed here:


